# Effect of estrogens on boar sperm capacitation *in vitro*

**DOI:** 10.1186/1477-7827-8-87

**Published:** 2010-07-13

**Authors:** Lukas Ded, Pavla Dostalova, Andriy Dorosh, Katerina Dvorakova-Hortova, Jana Peknicova

**Affiliations:** 1 Laboratory of Diagnostics for Reproductive Medicine, Institute of Biotechnology, Academy of Sciences of the Czech Republic, v. v. i., Prague, Czech Republic; 2 Department of Zoology, Faculty of Science, Charles University, Prague, Czech Republic

## Abstract

**Background:**

Mammalian sperm must undergo a series of controlled molecular processes in the female reproductive tract called capacitation before they are capable of penetrating and fertilizing the egg. Capacitation, as a complex biological process, is influenced by many molecular factors, among which steroidal hormone estrogens play their role. Estrogens, present in a high concentration in the female reproductive tract are generally considered as primarily female hormones. However, there is increasing evidence of their important impact on male reproductive parameters. The purpose of this study is to investigate the effect of three natural estrogens such as estrone (E1), 17beta-estradiol (E2) and estriol (E3) as well as the synthetical one, 17alpha-ethynylestradiol (EE2) on boar sperm capacitation in vitro.

**Methods:**

Boar sperm were capacitated in vitro in presence of estrogens. Capacitation progress in control and experimental samples was analyzed by flow cytometry with the anti-acrosin monoclonal antibody (ACR.2) at selected times of incubation. Sperm samples were analyzed at 120 min of capacitation by CTC (chlortetracycline) assay, immunocytochemistry and flow cytometry with anti-acrosin ACR.2 antibody. Furthermore, sperm samples and capacitating media were analyzed by immunocytochemistry, ELISA with the ACR.2 antibody, and the acrosin activity assay after induced acrosomal reaction (AR).

**Results:**

Estrogens stimulate sperm capacitation of boar sperm collected from different individuals. The stimulatory effect depends on capacitation time and is highly influenced by differences in the response to estrogens such as E2 by individual animals. Individual estrogens have relatively same effect on capacitation progress. In the boar samples with high estrogen responsiveness, estrogens stimulate the capacitation progress in a concentration-dependent manner. Furthermore, estrogens significantly increase the number of acrosome-reacted sperm after zona pellucida- induced acrosomal reaction.

**Conclusions:**

We demonstrate here the stimulatory effect of four different estrogens on boar sperm capacitation in vitro. According to our results, there is significant difference in the response to tested estrogens at different capacitation time and among individual animals. In animals with a high response to estrogens, there is a concentration-dependent stimulation of capacitation and individual estrogens have relatively the same effect. Effects of individual estrogens, differences in the response to them by individual animals, their time and concentration-dependent outcomes further contribute to our knowledge about steroidal action in sperm.

## Background

Capacitation involves the physiological changes that spermatozoa must undergo in the female reproductive tract or *in vitro *to obtain the ability to penetrate and fertilize the egg [1-3]. Capacitation is a complex molecular process that results in changes of calcium concentration, protein phosphorylation, acrosomal matrix and membrane rearrangement. As a complex biological process, capacitation can be influenced by many molecular factors in the uterine and oviductal fluid [4] and the effect of uterine and oviductal fluids depends on the specific stages of the estrous cycle [5]. Although capacitation naturally occurs in the female reproductive tract, it can be also performed *in vitro *using specific media and physical conditions [6,7].

Estrogens are a group of steroid compounds, named for their importance in the estrous cycle. Although estrogens have been considered mainly female reproductive hormones, they also play an important role in regulating male reproductive functions. The main breakthrough in this field was brought forth by estrogen receptor knock-out mice. Phenotypically, these mice have significant alteration in testes histology, spermiogenesis and they suffer from infertility [8].

In somatic cells, estrogens act through three known estrogen receptors - ERa, ERb and GPR30. ERa and ERb are called - classical estrogen receptor. They bind specific loci in DNA (estrogen response elements) and act as transcriptional factors. Recently, there has been evidence of a nongenomic effect of these receptors [9] and this effect may be important for estrogen regulation of the sperm function since sperm are supposed to be transcriptionally silent. Classical estrogen receptors were found in human spermatozoa and there is evidence for their direct interaction with phosphatidylinositol-3-OHKinase/Akt pathway [10]. This observation is important, because some receptors in sperm membrane are supposed to have only a passive role [11]. Classical estrogen receptors were recently found together with the aromatase and androgen receptor in pig spermatozoa [12]. Beside classical receptors, estrogens can act through the membrane estrogen receptor GPR30. GPR30 signalization is accompanied by calcium mobilization, therefore, a signalization through this receptor seems to be a good candidate for estrogen pathway in sperm. However, to this date there is no evidence for the presence of this receptor in the sperm. Finally, there is some evidence for the presence of putative estrogen receptors in the sperm, which is different from the classical ones. The antibodies against these putative receptors block the stimulatory effect of estrogens but their functions remain to be elucidated [13].

Although several studies report effects of estrogen in mature spermatozoa, there are some contradictory results in this field. There are a few papers from 1970 s - 1980 s concerning the effect of estrogens and progesterone on capacitation of hamster and rabbit sperm *in vivo *and *in vitro*. Gwatkin and Williams reported an inhibitory effect of the follicular fluid enriched by progesterone and estrogens on capacitation of rabbit spermatozoa *in vitro *[14]. Briggs obtained similar results with hamster sperm [15]. Contrary to this, Bathla et al. reported a significantly higher number of spermatozoa incubated in isolated uterus enriched by exogenous estrogens [16]. Further, Hamner and Wilson concluded that antiestrogens have no effect on the capacitation progress of rabbit sperm [17]. Recently, it was reported that there is a stimulatory effect of estrogens and different xenoestrogens on capacitation, acrosome reaction and fertilizing ability of mouse spermatozoa [18]. Furthermore, pre-incubation with estrogens does not alter the ability of human sperm to fuse with the oocyte [19].

In this study, we investigated the effect of three natural estrogens such as estrone (E1), 17β-estradiol (E2), estriol (E3), and one syntetical estrogen (17α-ethynylestradiol, EE2) on capacitation and AR of boar sperm *in vitro*.

## Methods

### Chemicals

All chemicals were purchased from Sigma (Prague, Czech Republic) unless otherwise specified.

### Sperm capacitation *in vitro *and calcium ionophore/zona pellucida-induced acrosomal reaction

Boar (Sus scrofa) ejaculates were supplied by Insemination Station, Kout na Sumave, CR. All sperm samples were examined for their motility and viability. Samples of poor quality were discarded. Suitable sperm samples were washed twice in tris-buffered saline (TBS, 200 × g, 10 min), centrifuged on Percoll gradient (80, 70, 55, 40% Percoll, 200 × g, 60 min) and washed in capacitation medium without bovine serum albumine (11.3 nM NaCl, 0,3 mM KCl, 1 mM CaCl_2_, 2 mM TRIS, 1.1 mM glucose, 0.5 mM pyruvate). After being washed and percolled, sperm were resuspended in capacitation medium (11.3 nM NaCl, 0.3 mM KCl, 1 mM CaCl_2_, 2 mM TRIS, 1.1 mM glucose, 0.5 mM pyruvate, BSA 1 mg/ml, pH 7.4) to concentration 5 × 10^7 ^sperm/ml. Experimental sperm samples were treated by estrogens to final concentrations 1 nM - 100 μM and control samples with the same amount of ethanol as in the experimental samples. Sperm suspension was incubated for the relevant time (30, 60, 90, 120, 180, 240 min) under paraffin oil at 37°C, 5% CO_2_. After 240 min of incubation, selected samples were treated by boar solubilized zona pellucida (ZP) (Czech Univerzity of Life Sciences, Prague, Czech Republic) for 30 min (37°C, 5% CO_2_).

### CTC assay

**Figure 1:**
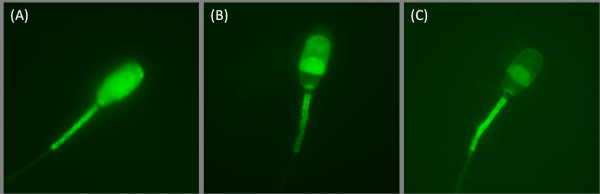
**CTC acrosomal fluorescent patterns**. Representative pictures of three specific CTC acrosomal fluorescent patterns. (A) Uncapacitated, acrosome intact sperm - bright fluorescence over the entire sperm head and positive mid-piece of the tail; (B) Capacitated, acrosome-intact sperm - prominent fluorescent positive equatorial segment and mid-piece of the tail, fluorescence-free (dark) band in the post-acrosomal region; (C) Acrosome-reacted sperm - low fluorescent signal throughout the sperm head, with a remaining positive signal in the equatorial segment and mid-piece.

The chlortetracycline (CTC) fluorescence assay was described previously [20,21]. After the capacitation process, sperm suspensions were centrifuged: the capacitation medium was removed and refrigerated for biochemical assays. Sperm were re-suspended in phosphate-buffered saline (PBS) and mixed with equal volume (45 μl/45 μl) of CTC solution (750 mmol/l CTC in 130 mmol/l NaCl, 5 mmol/l cysteine, 20 mmol/l Tris-HCl, pH 7.8) and incubated for 30 min. Cells were then fixed by 8 μl of 12.5% paraformaldehyde in 0.5 mol/l Tris-HCl (pH 7.4). After incubation, sperm suspension was placed on a glass slide, smeared and overlaid by a cover slip. To avoid evaporation and CTC fading, slides were kept in a wet chamber until the evaluation was carried out. Samples were examined with a Nikon Labothot-2 fluorescent microscope equipped with a 40× Nikon Plan 40/0.65 and photographed with a COHU 4910 CCD camera (Inc. Electronics Division, San Diego, USA) with the LUCIA imaging software (Laboratory Imaging Ltd., Prague, Czech Republic). Sperm were classified according to their acrosomal staining patterns: (A) Bright fluorescence over the entire sperm head and positive mid-piece of the tail - uncapacitated, acrosome intact sperm; (B) Prominent fluorescent positive equatorial segment, mid-piece of the tail and fluorescence-free (dark) band in the post-acrosomal region - capacitated, acrosome-intact sperm; (C) Low fluorescent signal throughout the sperm head, with remaining positive signal in the equatorial segment and mid-piece - acrosome-reacted sperm (Fig. 1). Sperm with a nonspecific or intermediate fluorescent signal status were not selected for subsequent analysis. In each sample, 200 cells were evaluated and the minimal number of evaluated samples was 5.

### Indirect immunofluorescence with anti-acrosin ACR.2 monoclonal antibody

**Figure 2:**
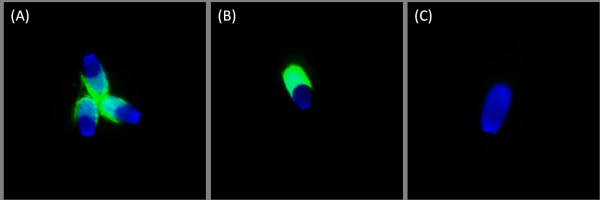
**ACR.2 Acrosomal fluorescent patterns (FITC-conjugated secondary antibody)**. Representative pictures of three specific ACR.2 acrosomal fluorescent patterns. (A) Uncapacitated, acrosome intact sperm - moderate uniform fluorescence in the acrosomal area; (B) Capacitated, acrosome-intact sperm - intensive fluorescence of the acrosome; (C) Acrosome-reacted sperm - low or no fluorescent signal in the sperm head. Nuclei stained with a Blue DAPI dye.

ACR.2 immunofluorescent analysis was described previously [22,23]. After the capacitation process, sperm suspensions were centrifuged; the capacitation medium was removed, and kept at -20°C. Sperm were re-suspended in equal volume of phosphate-buffered saline (PBS), smeared onto glass slides, dried and kept at 4°C. During fluorescent specimen preparation, sperm slides were fixed with acetone for 10 min, rinsed with PBS, treated with ACR.2 monoclonal antibody and incubated in a wet chamber for 60 min at 37°C. After thorough washing in PBS, the smears were treated with FITC-conjugated anti-mouse IgG antibody (Sigma, Prague, Czech Republic) and again incubated in a wet chamber for 60 min at 37°C. After washing in PBS and water, smears were mounted by the Vectashield mounting medium with DAPI (Vector Lab., Burlingame, CA). Samples were examined with a Nikon Labothot-2 fluorescent microscope equipped with 40× Nikon Plan 40/0.65 and photographed with a COHU 4910 CCD camera (Inc. Electronics Division, San Diego, USA) using LUCIA imaging software (Laboratory Imaging Ltd., Prague, Czech Republic). Sperm were classified according to their acrosomal staining patterns. (A) Moderate fluorescence in the acrosomal area - uncapacitated, acrosome intact sperm; (B) Intensive fluorescence of the acrosome -- capacitated, acrosome-intact sperm; (C) Low or no fluorescent signal in the sperm head with a remaining positive equatorial segment - acrosome-reacted sperm (Fig. 2). Sperm with nonspecific or intermediate acrosomal status were not selected for subsequent analysis. In each sample, 200 cells were evaluated and the minimal number of evaluated samples was 5.

### Flow cytometry analysis with ACR.2 antibody

The control, capacitated and experimental sperm samples from animals with high responsiveness to E (animal A) were influenced by 1 μM E2, then washed in PBS and fixed by 96% ethanol at 4°C for 60 min. After ethanol fixation, sperm were refixed in ethanol-acetone mixture at 4°C (1:1) for 30 min. Sperm were then washed three times in PBS and incubated with anti-acrosin ACR.2 antibody (50 μg/ml) at 37°C for 60 min. After the incubation with the primary antibody, sperm were washed three times in PBS and incubated with a secondary anti-mouse IgG antibody (Sigma, Prague, Czech Republic). After the incubation sperm samples were intensively washed in PBS (five times for 5 min) then 100 μl of the suspension was placed on 96-well plate. Acquisition and analysis were performed on BD LSR II instrument (BD, Becton Drive Franklin Lakes, NJ, USA), excitation laser 488 nm, emission filters 530/40, measurement of fluorescent intensity in FITC channel. Analysis was performed using FlowJo 7.5.4. software (TreeStar Inc., Ashland, OR, USA). The differences among control and experimental samples in arithmetic mean of the fluorescent intensity in the FITC channel were assessed.

### Indirect ELISA with ACR.2 antibody

After *in vitro *capacitation, sperm samples were centrifuged and sperm-free capacitating medium was collected for subsequent biochemical analysis. Capacitation medium was lyophilised and dissolved in determined volume of water. 100 μl of the dissolved lyophilisate was applied on a microtiter plate and incubated for 24 hours. After one-day of incubation, the plate was washed three times by PBS and PBS-TWEEN (2%). The cells were treated by ACR.2 monoclonal antibody [22] and incubated for 60 min. After incubation with primary ACR.2 antibody, the plate was washed and treated with peroxidase-conjugated swine anti-mouse antibody (SWAM-Px, Sevapharma, Prague, Czech Republic) conjugated and incubated for 30 min. After the second incubation, the plate was washed, and cells were treated by *o*-phenylenediamine (Fluka, Buchs, Switzerland) for 3 min. The reaction was stopped by 4N sulfuric acid and the absorbance was measured on Biotrak II Plate Reader (Amersham Biociences) at 492 nm.

### Acrosin activity assay

After the *in vitro *capacitation process, sperm samples were centrifuged and sperm-free capacitating medium was collected for subsequent analysis. Capacitation medium was lyophilised and redissolved in 100 μl of reaction buffer (0.2 M Tris.HCl, 0.02 M CaCl2, pH = 8), placed on a microtiter plate and incubated for 10 min. After the first incubation the BAPA solution (1 mg Nα-Benzoyl-L-arginine 4-nitroanilide hydrochloride/1 ml dimethylformamide) was added and this was incubated for 20 min. After the second incubation, the reaction was stopped by 30% formic acid and the absorbance of samples was measured on a Biotrak II Plate Reader (Amersham Biociences) at 405 nm.

### Statistical analysis

Experimental data were analyzed using STATISTICA 7.0. (StatSoft CR, Prague, Czech Republic). The statistical differences in the number of sperm with specific acrosomal status among control and experimental samples were assessed by the Kruskal-Wallis one-way analysis of variance (KW-ANOVA). Statistical differences between the continuous values (arithmetic means of the fluorescent intensity in the FITC channel in flow cytometry analysis, absorbance in indirect ELISA with ACR.2 antibody and acrosin activity assay) were assessed by one-way analysis of variance ANOVA. Post hoc analysis was performed by the Newman-Keuls test and multiple comparisons of mean ranks. The P value, *P < 0.05, **P < 0.01, ***p < 0.001.

## Results

### ACR.2 flow cytometry analysis of 1 μM E2 effect on capacitation at selected capacitation times

**Figure 3:**
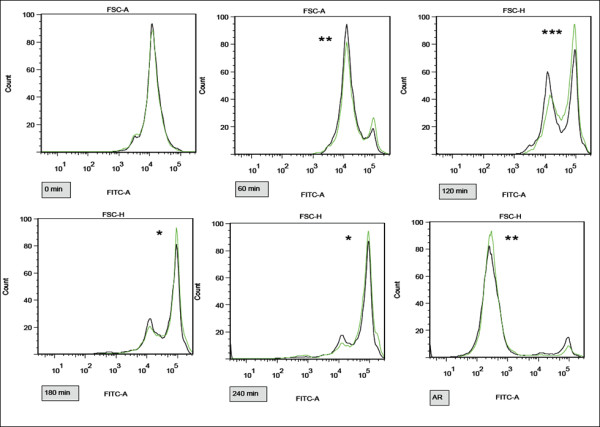
**Differences in capacitation and AR progress between samples A-D exposed by 1 μM E2 and control samples measured by flow cytometry with ACR.2 antibody**. Representative pictures of FITC channel histograms at 0, 60, 120, 180, 240 min and after induced AR. Control samples in black, experimental samples in green. The increase in fluorescent intensity (right peak) corresponds to the capacitation progress. Differences among the control and experimental samples in arithmetic mean of the fluorescent intensity in the FITC channel were assessed by t-test. *P < 0.05, **P < 0.01, ***P < 0.001. The most significant difference is recognizable at 120 min.

In order to determine the potential differences in the capacitation progress between control and experimental groups, sperm samples were analyzed by flow cytometry with ACR.2 antibody at selected times of capacitation. The experimental sample was capacitated with E2 at a 1 μM concentration, and the control sample with the same amount of ethanol as in the experimental sample. 1 μM concentration of E2 was selected based on the previous mouse study [18] where it was defined as the lowest concentration with any significant effect on sperm capacitation. Sperm were collected at 0, 10, 30, 60, 120, 180, and 240 min of capacitation and after the induced acrosomal reaction. The first significant difference between the control and experimental group was at 60 min capacitation in the arithmetic mean of the fluorescent intensity in the FITC channel (Fig. 3). The strongest significant difference was then at 120 min of capacitation. After an induced acrosomal reaction, a significantly higher number of sperm underwent AR in the experimental group in comparison with the control one.

### **Analysis of 1 μM E2 effect on capacitation by CTC fluorescence assay and anti-acrosin ACR.2 monoclonal antibody**

**Figure 4:**
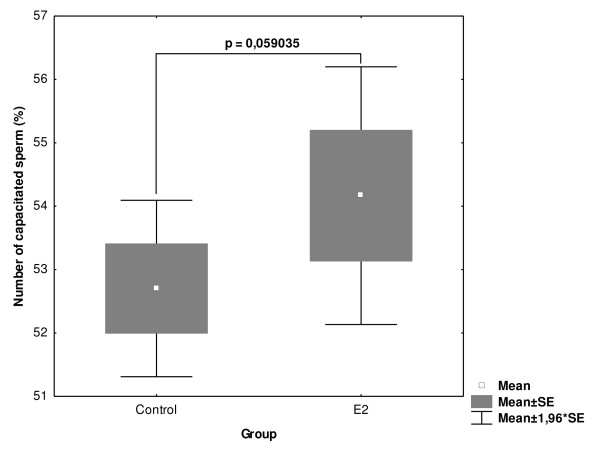
**Differences in the number of capacitated sperm in control and experimental samples influenced by 1 μM E2 in 8 individual animals**. Sperm samples from 8 individual animals were capacitated with 1 μM E2 and ethanol (control). Sperm were collected after 120 min of capacitation and analyzed by CTC and ACR.2 immunofluorescence. Differences were analyzed by Mann-Whitney U test *P < 0.05, **P < 0.01, ***P < 0.001.

**Figure 5:**
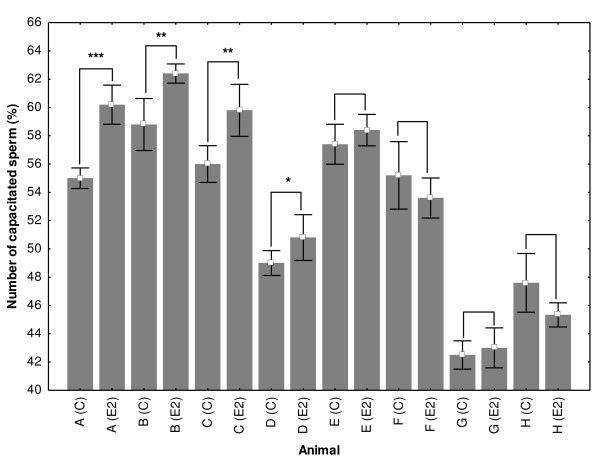
**Differences in the capacitation progress and response to estrogen (E2) among individual animals**. Animals A-H. Control samples (A (C) - H (C) and samples incubated with 1 μM E2 (A (E2) - H (E2). Whiskers denote ± SE. Differences were analyzed by Mann-Whitney U test, *P < 0.05, **P < 0.01, ***P < 0.001.

Sperm samples from 8 animals were capacitated in parallel, in the presence of experimental concentration of 1 μM E2 and ethanol (control) collected after 120 min of capacitation and analyzed by CTC and ACR.2 immunofluorescence. Only highly correlated results were used in the subsequent statistical analysis. E2 has almost significant (p = 0.059) procapacitation effect on boar sperm (Fig. 4). In order to evaluate the potential differences in the capacitation progress and the responsiveness to E2 among individual animals the samples from each boar were analyzed separately. There were significant differences in the capacitation progress and responsiveness to estrogens among individual animals (Fig. 5). In 4 samples, E2 significantly increased the number of capacitated sperm; in 4 other samples, E2 had no significant effect on the capacitation progress.

### CTC and ACR.2 analysis of the different estrogen-concentration effect at 120 min capacitation in samples with high response to E2 (boar A) and no significant response to 1 μM E2 (boar E)

Since the most significant difference between the control and E2-influenced experimental group was at 120 min of capacitation, sperm samples from boar A (with a high responsiveness to E2) and from boar E (with no significant response to 1 μM E2) were capacitated with a different concentration of four estrogens and analyzed by CTC and immunocytochemistry with ACR.2. In each group, at least 5 samples were analyzed. In CTC assay and immunocytochemistry with ACR.2 antibody, only highly correlated results (difference < 5%) were used in the subsequent statistical analysis. In the boar A sample, all estrogens significantly accelerate the capacitation progress in a concentration-dependent manner (Tab. 1). E2 showed the first significant effect at 10 nM concentration. All selected estrogens significantly accelerate the capacitation progress at 100 nM concentration. In sperm samples from boar E, only high concentrations of estrogens (10-100 μM) stimulated the capacitation progress (Tab. 2).

**Table 1:** Number of capacitated sperm in control and experimental samples after 120 of capacitation

Group	Control	1 nM	5 nM	10 nM	100 nM	1 μM	10 μM
**E1**	55.00 ± 1.56	55.5 ± 0.93	55.45 ± 1.79	56.25 ± 1.28	57.60 ± 1.26**	59.75 ± 1.58***	61.50 ± 0.93***
**E2**	55.00 ± 1.56	56.00 ± 1.41	56.74 ± 1.21*	56.80 ± 1.62*	57.73 ± 1.27***	60.20 ± 1.93***	62.00 ± 1.41***
**E3**	55.00 ± 1.56	54.88 ± 1.36	55.14 ± 1.75	56.14 ± 1.35	57.00 ± 0.89**	59.78 ± 1.48***	61.71 ± 0.76***
**EE2**	55.00 ± 1.56	55.44 ± 1.81	54.99 ± 2.25	55.80 ± 1.93	56.82 ± 2.04*	58.30 ± 2.91**	62.00 ± 1.41***

Sperm samples from boar A (with high responsiveness to E2) were capacitated in the presence of six different concentrations (1 nM - 10 μM) of four estrogens and analyzed by CTC and immunocytochemistry with ACR.2. In each group, at least 5 samples were analyzed. In CTC assay and immunocytochemistry with ACR.2 antibody, only highly correlated results (difference < 5%) were used in the subsequent statistical analysis. All estrogens significantly accelerated the capacitation progress in a concentration-dependent manner. E2 had the first significant effect at 10 nM concentration, other three estrogens at 100 nM concentration. Differences were analyzed by KW-ANOVA; post hoc comparison was performed by multiple comparisons of mean ranks. *P < 0.05, **P < 0.01, ***P < 0.001.

**Table 2:** Number of capacitated sperm in control and experimental samples after 120 of capacitation

Group	Control	1 nM	10 nM	100 nM	1 μM	10 μM	100 μM
**E1**	57.4 ± 1.14	57.5 ± 1.89	57.55 ± 1.78	57.37 ± 2.62	58.11 ± 1.58	60.12 ± 1.12**	61.25 ± 1.56***
**E2**	57.4 ± 1.14	58.00 ± 1.61	58.80 ± 1.62	58.11 ± 2.11	58.40 ± 0.89	60.10 ± 1.22**	61.81 ± 1.22***
**E3**	57.4 ± 1.14	56.89 ± 1.75	56.14 ± 1.35	57.00 ± 1.76	57.78 ± 2.45	60.22 ± 1.56**	61.54 ± 1.67***
**EE2**	57.4 ± 1.14	57.84 ± 2.09	57.70 ± 1.93	58.42 ± 1.36	58.30 ± 1.98	60.10 ± 2.31**	61.43 ± 1.37***

Sperm samples from boar E (with no significant responsiveness to E2 at concentration of 1 μM) were capacitated in the presence of six different concentrations (1 nM - 100 μM) of four estrogens and analyzed by CTC and immunocytochemistry with ACR.2. In each group, at least 5 samples were analyzed. In CTC assay and immunocytochemistry with ACR.2 antibody, only highly correlated results (difference < 5%) were used in the subsequent statistical analysis. All estrogens significantly accelerated the capacitation progress at 10 μM concentration. Differences were analyzed by KW-ANOVA; post hoc comparison was performed by multiple comparisons of mean ranks. *P < 0.05, **P < 0.01, ***P < 0.001.

### Analysis of the differences in the number of sperm after ZP-induced AR incubated with 1 μM estrogens

**Figure 6:**
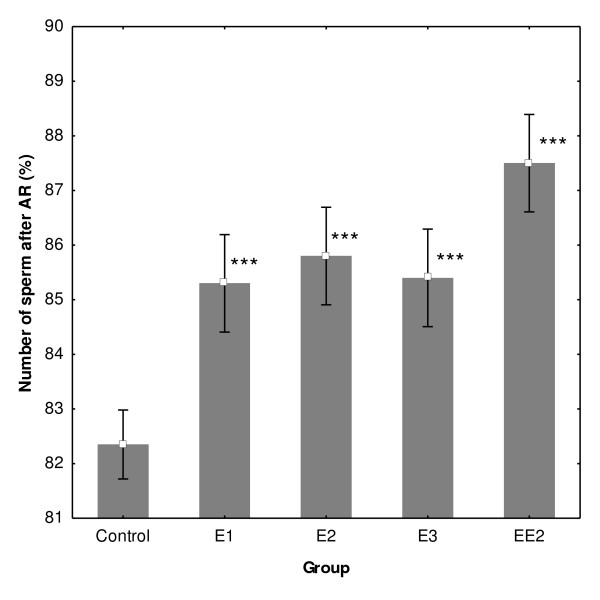
**Number of sperm after AR evaluated by ACR.2 antibody**. After 240 min of capacitation, control and experimental samples from boars A-D with 1 μM concentration of four estrogens were treated by zona pellucida to induce AR. There was a significantly higher number of sperm, which underwent calcium ZP-induced AR in all experimental samples in comparison to the experimental group. All estrogens significantly increased the number of sperm after the ZP-AR. Differences were analyzed by KW-ANOVA; post hoc comparison was performed by multiple comparisons of mean ranks. Whiskers denote ± SE. *P < 0.05, **P < 0.01, ***P < 0.001.

**Figure 7:**
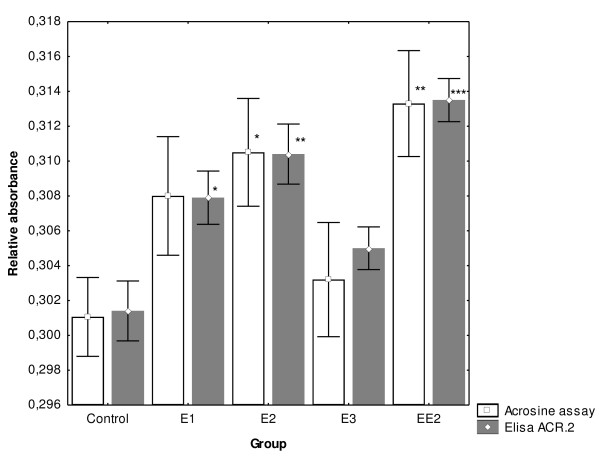
**Concentration of acrosin in medium after induced AR measured enzymatically by acrosin assay and immunochemically by ELISA with ACR.2 antibody**. After 240 min of the *in vitro *capacitation process, experimental samples from boars A-D with 1 μM concentration of four estrogens were treated by boar zona pellucida to induce AR. Capacitation media were analyzed by ELISA with ACR.2 antibody and acrosin assay to biochemically determine the number of sperm after AR. Differences were analyzed by ANOVA; post hoc comparison was performed by Newman-Keuls test. Whiskers denote ± SE. *P < 0.05, **P < 0.01, ***P < 0.001.

Sperm samples were analyzed after 240 min of capacitation and induced AR by CTC, immunocytochemistry and ELISA with ACR.2 antibody. The acrosin assay was used to further evaluate the effect of estrogens on capacitation and the acrosomal reaction. There was a significantly higher number of sperm, which underwent ZP -induced AR in all experimental samples in comparison with the experimental group (Fig. 6). Data from immunocytochemistry were further verified by ELISA with ACR.2 antibody and the acrosin assay (Fig. 7).

## Discussion

In this study, we addressed several questions concerning the effect of estrogens on boar sperm *in vitro*. Although several previous studies have reported on the effects of estrogen in mature spermatozoa among different species, there are some contradictory results in this field. Therefore we employed multiple evaluation techniques to complexly analyze the effect of estrogen on boar sperm *in vitro*. The obtained results from each method might be useful whilst searching for specific mechanisms, which mediate the estrogen effect in mammalian sperm.

The first experiment addressed the question of whether strong, naturally occurring estrogen (E2) has a significant impact on the boar sperm capacitation progress at different capacitation times. Sperm were capacitated *in vitro *in the presence of 1 μM E2 or ethanol (control). We found out that 1 μM E2 has a procapacitation effect on the boar sperm *in vitro*. Furthermore, we demonstrated the non-identical effect of E2 on capacitation at different times of incubation. The first significant difference between the control and experimental samples was at 60 min, and the strongest response was at 120 min of capacitation. In the later capacitation stages 180 min onwards, the difference between the control and experimental samples was not significant. The observed time-dependent effect of estrogens on the capacitation process is an important finding. In previous publications, authors analysed sperm capacitation status after 30 min [18], 180 - 300 min [16] and 360 min [14] and this fact might be an important source for some of the contradictory results. Therefore, the effect of estrogens on capacitation of mammalian sperm should be analyzed at carefully selected capacitation times reflecting the status of the ongoing sperm capacitation process in individual species. Furthermore, the analysis of the time-dependent effect of estrogens on capacitation might be useful while searching for specific molecular processes, which are temporally correlated with the most significant effect of estrogens (e.g. calcium influx, cholesterol efflux, actin polymerisation, protein phosphorylation, acrosomal rearrangement etc.) [24].

In the second experiment, we wondered whether E2 has a similar effect on sperm samples collected from different individuals. We observed strong differences in the response to estrogens among samples from different individual animals during capacitation *in vitro*. According to our results, the analysis of different responsiveness to estrogens among individual animals in the tested population might be important, because individual variability strongly affects general results. Furthermore, a detailed analysis of the individuals with high and low estrogen-responsiveness can elucidate the mechanism of the estrogen action in sperm. Hitherto, there is no plausible parameter e.g. expression of a different estrogen receptor correlating with estrogen responsiveness [25].

In the third experiment, we tested the effect of four different estrogens on the capacitation progress of sperm collected from boar with high and no significant difference. Analysis of the effect of multiple compounds with a similar physiological effect (E1, E2, E3, EE2) provides more reliable data than analysis based only on one compound. In an animal with high estrogen-responsiveness, estrogens stimulate capacitation in a concentration dependent manner. E2 has a significant effect at 5 nM concentration; all other estrogens have a significant effect at 100 nM concentration. Although contrary to other estrogens, E2 had a significant effect at 5 nM and 10 nM concentrations. There was no significant difference between individual estrogens at the appropriate concentration level. In the boar sample with no significant response to 1 μM E2, only a very high concentration of estrogens stimulates capacitation (10-100 μM). It suggests that estrogens have a general procapacitation effect, but in some animals, the responsiveness to estrogens is low and only very high concentrations of estrogens are able to provoke a procapacitation effect. The differences in estrogen-responsiveness further suggest that multiple mechanisms in the estrogen action in sperm might be involved. In the samples with high responsiveness, estrogens (E2) have a significant effect at concentrations normally required for estrogen receptor-mediated cellular response [26,27]. The fact that a higher concentration of estrogens at which estrogen receptors are almost saturated will still increase the number of capacitated cells in a concentration-dependent manner suggests that the estrogen effect at high concentration might be mediated by another, nonreceptor mechanism (membrane changes, etc.) [25]. This idea is further supported by the fact that in samples with no significant response to 1 μM E2, estrogens have a significant effect at high concentrations (10-100 μM), which are far from a concentration needed for the estrogen receptor mediated cellular response in somatic cells. This fact suggests that the specific mechanism (e.g. receptor signalization), which is responsible for estrogen responsiveness at low concentration, is not functional in samples with no response to 1-10 nM E2. Nevertheless, high experimental concentrations of estrogens (10-100 μM) are far from the physiological plasma levels of estrogens (e.g. 10^-10^-10^-11^M for E2 in rats and mice [28]). However, the concentration of estrogens in follicular fluid is on the other hand higher [28] and sperm may be, therefore, exposed to high concentrations during their capacitation in the female reproductive tract [29,30].

Finally, in the last experiment, we demonstrated the significant impact of estrogens on the ZP-induced acrosomal reaction. The number of sperm after AR was significantly higher in all experimental groups. The induced acrosomal reaction data were evaluated microscopically and also confirmed by objective biochemical methods. Therefore, the results obtained from ZP-induced acrosomal reaction, not only confirm the capacitation experiment, but also suggest that estrogens have a real physiological impact on sperm capacitation, as the analysis was based, in particular, on molecular and cellular markers of capacitation (calcium influx, acrosomal rearrangement). Furthermore, the analysis by objective biochemical methods (ELISA, acrosin assay) provides an important supporting data to the subjective microscopical evaluation methods.

In conclusion, in this study we addressed several important questions concerning the effect of estrogens on boar sperm capacitation *in vitro*. We found out that in boar sperm *in vitro *estrogens generally show a procapacitation effect. This effect depends strongly on the stage of the capacitation progress, estrogen concentration and individual responsiveness of tested animals. Individual estrogens have a relatively similar effect. These observations have a significant impact on our understanding of the previous results concerning estrogen effects in sperm and should be helpful to uncover the specific mechanisms of the estrogen effects in sperm physiology.

## Competing interests

The authors declare that they have no competing interests.

## Authors' contributions

LD is responsible for sperm capacitation, analysis of the capacitation process by all described methods, for statistical analysis and preparation of the manuscript. PD is responsible for the preparation of the media with specific concentrations of estrogens, sperm capacitation, analysis of sperm capacitation status by CTC and immunocytochemistry by ACR.2 antibody. AD is responsible for sperm capacitation and analysis of capacitation media after the induced AR. JP and KH are responsible for the conception of the study, coordination of experiments, revising and final approval of the manuscript. All authors read and approved the final manuscript.
